# Development and Validation of Ferroptosis-Related LncRNA Biomarker in Bladder Carcinoma

**DOI:** 10.3389/fcell.2022.809747

**Published:** 2022-03-02

**Authors:** Yiru Wang, Shijie Zhang, Yang Bai, Gen Li, Siyu Wang, Jiayi Chen, Xin Liu, Hang Yin

**Affiliations:** ^1^ Department of Gynecologic Oncology, Harbin Medical University Cancer Hospital, Harbin, China; ^2^ Department of Radiation Oncology, Harbin Medical University Cancer Hospital, Harbin, China; ^3^ Department of Pharmacology, State-Province Key Laboratories of Biomedicine-Pharmaceutics of China, Ministry of Education, College of Pharmacy, Harbin Medical University, Harbin, China; ^4^ Key Laboratory of Cardiovascular Medicine Research, Ministry of Education, College of Pharmacy, Harbin Medical University, Harbin, China

**Keywords:** bladder cancer, long non-coding RNA, immune, ferroptosis, biomarkers

## Abstract

Bladder cancer (BC) is a highly prevalent cancer form of the genitourinary system; however, the effective biomarkers are still ambiguous and deserve deeper investigation. Long non-coding RNA (lncRNA) occupies a prominent position in tumor biology and immunology, and ferroptosis-related genes participate in regulatory processes of cancer. In this study, 538 differentially expressed ferroptosis-related lncRNAs were identified from the The Cancer Genome Atlas database through co-expression method and differential expression analysis. Then, the samples involved were equally and randomly divided into two cohorts for the construction of gene model and accuracy verification. Subsequently, a prediction model containing five ferroptosis-related lncRNAs was constructed by LASSO and Cox regression analysis. Furthermore, in terms of predictive performance, consistent results were achieved in the training set, testing set, and entire set. Kaplan–Meier curve, receiver operating characteristic area under the curve, and principal component analysis results verified the good predictive ability of model, and the gene model was confirmed as an independent prognostic indicator. To further investigate the mechanism, we explored the upstream of five lncRNAs and found that they may be modified by m6A to increase or decrease their expression in BC. Importantly, the low-risk group displayed higher mutation burden of tumors and lower Tumor Immune Dysfunction and Exclusion score, which may be predicted to have a higher response rate to immunotherapy. Interestingly, the patients in the high-risk group appeared to have a higher sensitivity to traditional chemotherapeutic agents through pRRophetic analysis. In general, our research established a five-ferroptosis-related lncRNA signature, which can be served as a promising prognostic biomarker for BC.

## Background

Bladder cancer (BC) is one of the main human malignant tumors, which has high morbidity and mortality. There were approximately 81,400 newly diagnosed cases and 17,980 deaths in United States in 2020 (Siegel et al., 2020). BC can be classified into muscle-invasive bladder cancer (MIBC) and non-muscle-invasive bladder cancer (NMIBC) according to the extent of tumor invasion (Thompson 2006). Approximately 75% of newly diagnosed urothelial BC is NMIBC (Burger et al., 2013). The main diagnostic modalities for NMIBC is transurethral resection and cystoscopy (Babjuk et al., 2017; Sefik et al., 2019). In addition, most patients are diagnosed as late-stage cancer with postoperative drug resistance and rapid progression (Dreicer 2017; Robertson et al., 2018). Because of the high recurrence rate of BC, continuous surveillance strategies and treatment were necessary (Svatek et al., 2014). The biomarkers related to early diagnosis and prognosis prediction are especially important and urgently need.

Whether immune escape or immune response, the occurrence and development of tumor is inseparable from the immunity. Immunotherapy has a good therapeutic effect in many tumors and brought hope to many patients with cancer (Sharma and Allison 2015). *Bacillus* Calmette Guerin (BCG) is the main drug for intravesical instillation of BC because of the enhanced immune response (Kamat et al., 2016). Nevertheless, tumor immune escape brings challenges to immunotherapy (Tang et al., 2020). It is necessary to analyze the relationship between BC and immunity.

Long non-coding RNA (lncRNA) is a special class of non-coding RNAs longer than 200 nucleotides in length (Mendell 2016). Many lncRNAs recruit regulatory protein complexes to regulate transcription (Engreitz et al., 2016). Our previous studies have shown that some lncRNAs were different from that in normal tissues, which could be prognostic factors in many cancers including head and neck squamous cell, clear cell renal, and endometrium (Yin et al., 2018; Xu et al., 2019; Wang et al., 2020). Furthermore, the lncRNA *LNMAT2* and *SNHG16* enhanced tumor lymphangiogenesis and lymph node (LN) metastasis, which may be attractive therapeutic targets for BC with LN metastasis (Chen et al., 2020; Chen C. et al., 2021). However, the role of lncRNAs in BC has not fully understood and merit further work.

As a new star in cancer, ferroptosis is a kind of programmed cell death differed from cell necrosis, apoptosis, and autophagy. The characteristic of ferroptosis is accumulation of heavy ROS and iron (Dixon et al., 2012). In addition, ferroptosis is closely related to tumor that affects tumor-related signaling pathways and plays an important role in chemotherapy, radiotherapy, and immunotherapy (Chen X. et al., 2021). There are many ferroptosis regulators consist of GPX4 (Yang et al., 2014), SLC7A11 (Koppula et al., 2021), ACSL4 (Doll et al., 2017), etc. According to many current works, lncRNAs may regulate the ferroptosis as an epigenetic regulator. *LINC00336* could regulate the expression of cystathionine-β-synthase and inhibit ferroptosis combined with ELAV-like RNA-binding protein 1 (ELAVL1) (Wang M. et al., 2019). The cytosolic *lncRNA P53RRA* activate the p53 pathway to promote ferroptosis and apoptosis (Jiang et al., 2015). Nevertheless, the regulator role of some ferroptosis-related lncRNAs in BC has not been fully understood.

Therefore, we analyzed BC genes from The Cancer Genome Atlas (TCGA) and screen out differentially expressed (DE) ferroptosis-prognosis–related lncRNAs. The five ferroptosis-prognosis–related lncRNAs were screened out. Furthermore, we built a prediction model that had been tested the strong prediction ability. The result of survival analysis confirmed that the risk score was inversely correlated with the survival of patients. Cox regression analysis showed that the risk score is an independent risk factor for BC. Then, we compared the differences in clinical stage, immunity, and methylation between the two risk groups and carried out depth immunoassay. Thus, it is confirmed that the five-ferroptosis-related lncRNA signature that closely related to tumor immunity has great significance for the prognosis prediction of BC.

## Materials and Methods

### Data Collection and lncRNAs Screening

We downloaded clinical and RNA sequencing data of BC from TCGA (https://portal.gdc.cancer.gov/). Samples with insufficient clinical information were excluded. Hence, there are 406 BC tissues and 19 normal tissues with mRNA sequencing and lncRNA sequencing. Ferroptosis genes were acquired from FerrDb (www.zhounan.org/ferrdb/). A total of 1,752 ferroptosis-related lncRNAs were selected by the co-expression analysis between ferroptosis genes and BC lncRNAs (|cor| > 0.4 and *p* < 0.001). We explored the significantly DE BC genes, ferroptosis genes, and ferroptosis-related lncRNAs between cancer and normal samples using the limma package. The cutoff value was |log2FC| > 1 and FDR <0.05 (FC, fold change; FDR, false discovery rate).

### Function and Pathway Enrichment of DE Ferroptosis Genes

We used the clusterProfler package to analyze the Gene Ontology (GO) and Kyoto Encyclopedia of Genes and Genomes (KEGG) pathways of DE ferroptosis genes to further explore the underlying molecular functions (MFs) and cellular components (CCs) (*p* < 0.05).

### Identification and Selection of Ferroptosis-Prognosis–Related lncRNAs

The ferroptosis-prognosis–related lncRNAs were screened by univariate Cox regression analysis. The LASSO regression analysis was used on the screened genes and establishes the risk model. Then, we studied the lncRNAs that can affect BC alone by multivariate regression analysis.

All 406 BC samples were randomly divided into two sets. The risk score for each sample was calculated using the formula $\text{Risk score }\left(\text{RS}\right)=\sum_{i=1}^{N}\left(Expi*Coei\right)\;$ (N is the number of prognostic lncRNA genes, Expi is the expression value of lncRNA, and Coei is the estimated regression coefficient of lncRNA in the multivariable Cox regression analysis), which could predict the prognosis risk of patients with BC. According to the median risk score, the patients in each group were divided into high- and low-risk groups. Overall survival (OS) was compared between the two risk groups. The receiver operating characteristic (ROC) was carried out with the R package to test predictive ability of our model. Principal component analysis (PCA) was used to explore the distribution of samples in different risk group. Survival analysis and Cox regression analysis were performed on the clinical variables (age, gender, grade, and stage) and the risk scores.

### Construct the Network of Ferroptosis-Prognosis–Related lncRNAs

To explore the connections of these ferroptosis-related lncRNAs that can predict prognosis of BC, co-expression analysis was performed to construct the network. We used the database to explore the interaction between and distinguished genes according to *p* value.

### Comparative Analysis of High- and Low-Risk Group

Previous studies have confirmed that methylation is of great significance in tumors (Yin et al., 2021). To further explore the relationship between our risk grouping, clinical variables and m6A methylation, we compared the differences in TNM staging system, age, gender, grade, stage, and m6A methylation genes between the high- and low-risk groups.

### Immunoassay

We used different algorithms to evaluate the immune cell abundance of the high- and low-risk groups including and studied the correlation between immune cell content and the risk score in BC samples. Furthermore, the differences of immune function, immune checkpoints, and molecular typing of immune subtypes between the two risk groups were studied. Moreover, to explore the significance of our risk model in immune escape and immunotherapy, we used VarScan to discuss the tumor mutation burden (TMB) and calculated the Tumor Immune Dysfunction and Exclusion (TIDE) score. To further explore the effect of immunity on BC, tumor samples were divided into two groups according to the quantity of memory B cells, T cells, macrophages, neutrophils, etc., and Kaplan–Meier survival curve was drawn. Similarly, we evaluated the immune function of BC samples and divided them into two groups according to the median immune score. Survival analysis was performed for different groups.

### Drug Sensitivity Analysis

In addition, drug resistance is a bottleneck in tumor treatment. To explore the relationship between the risk score and the sensitivity of antineoplastic agents, we used an R package to calculate the 50% inhibiting concentration (IC50) of commonly used drugs for BC.

### Statistical Analysis

R software package limma selected the DE genes between tumor tissues and normal tissues. LASSO regression analysis was carried out with glmnet package. We used Cox regression analysis to identify prognostic factors for BC. Survival curves were plotted by the survival package for R. To further confirmed prediction ability of the risk score, PCA was used to describe the distribution of the high- and low-risk groups.

## Results

### Differentially Expressed Gene in BC

**FIGURE 1:**
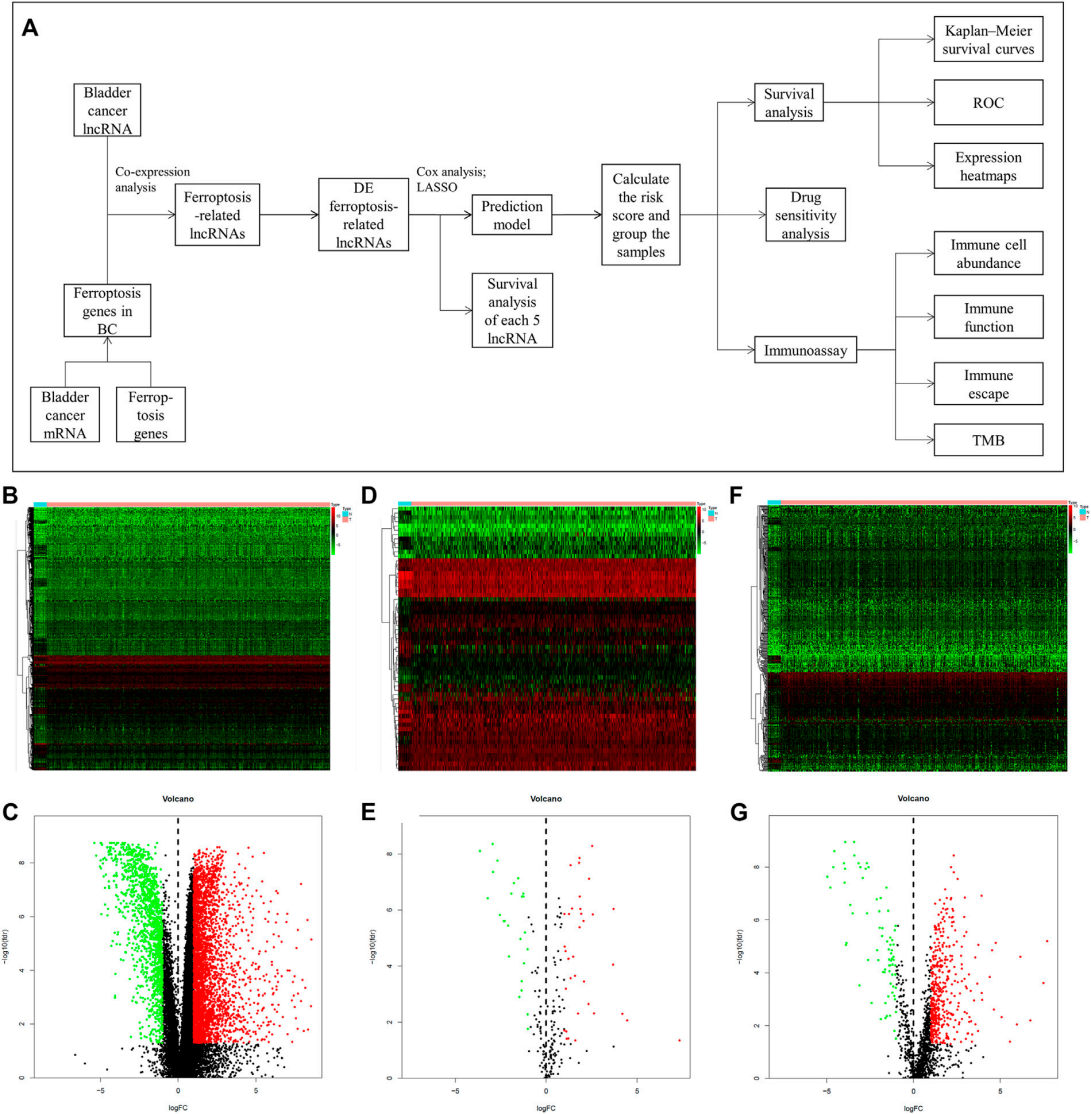
The mentality of this study and comparison of gene expression between bladder tissue and adjacent normal tissue. **(A)** Flow chart of this research. Expression heatmaps of bladder cancer genes including mRNA and lncRNA **(B)**, ferroptosis genes **(D)** and ferroptosis-related lncRNAs **(F)**. Volcano plot of differentially expressed bladder cancer genes **(C)**, ferroptosis genes **(E)** and ferroptosis-related lncRNAs **(G)** between normal and tumor samples.

The flow chart of this study is shown in Figure 1A. We first acquired the data from TCGA and identified 4,847 DE genes (including mRNAs and lncRNAs) of BC. There were 3,429 upregulated and 1,418 downregulated genes in tumor samples compared to normal samples (Figures 1B,C). In addition, 61 DE ferroptosis genes (36 elevated and 25 downregulated; Figures 1D,E) were distinguished. Co-expression analysis was used to find ferroptosis-related lncRNAs. Among them, 538 DE ferroptosis-related lncRNAs (463 elevated and 75 downregulated; Figures 1F,G) were screened out between tumor tissues and normal tissues (|log2FC| > 1 and FDR <0.05).

### GO and KEGG Pathway Enrichment Analysis

**FIGURE 2:**
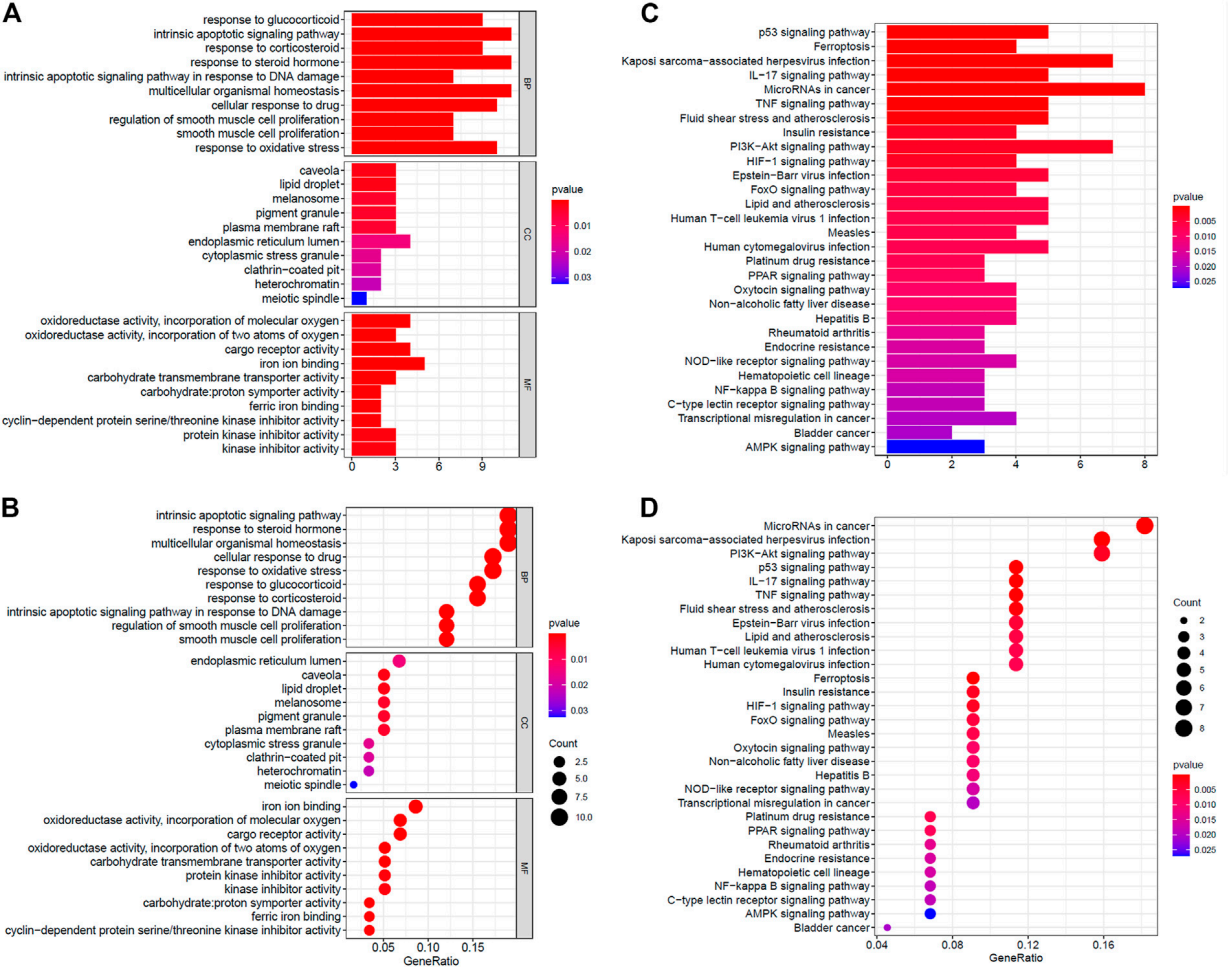
Functional enrichment analysis of ferroptosis-related genes. **(A,B)** The GO analysis of DE ferroptosis genes. **(C,D)** The KEGG analysis revealed that DE ferroptosis genes were associated with microRNAs, Kaposi’s sarcoma-associated herpesvirus infection, PI3K-Akt signaling pathway, etc.

The GO analysis revealed that DE ferroptosis genes is related to intrinsic apoptotic, multicellular organismal homeostasis, and response to some stimulus including steroid hormone, drug, and oxidative stress, at the biological process (BP) category. The relationship between some DE genes and BP is shown in Supplementary Figure S1A. For the MF category, the DE genes were involved in iron ion binding, oxidoreductase activity, acting on single donors with incorporation of molecular oxygen, cargo receptor activity, etc. What is more, they mainly enriched in endoplasmic reticulum lumen, caveola, lipid droplet, melanosome, pigment granule, etc., at the CC category (Figures 2A,B). The KEGG analysis revealed that DE ferroptosis genes were associated with microRNAs, Kaposi’s sarcoma-associated herpesvirus infection, PI3K-Akt signaling pathway, etc. (Figures 2C,D).

### Establish and Verify the Prediction Model

**FIGURE 3:**
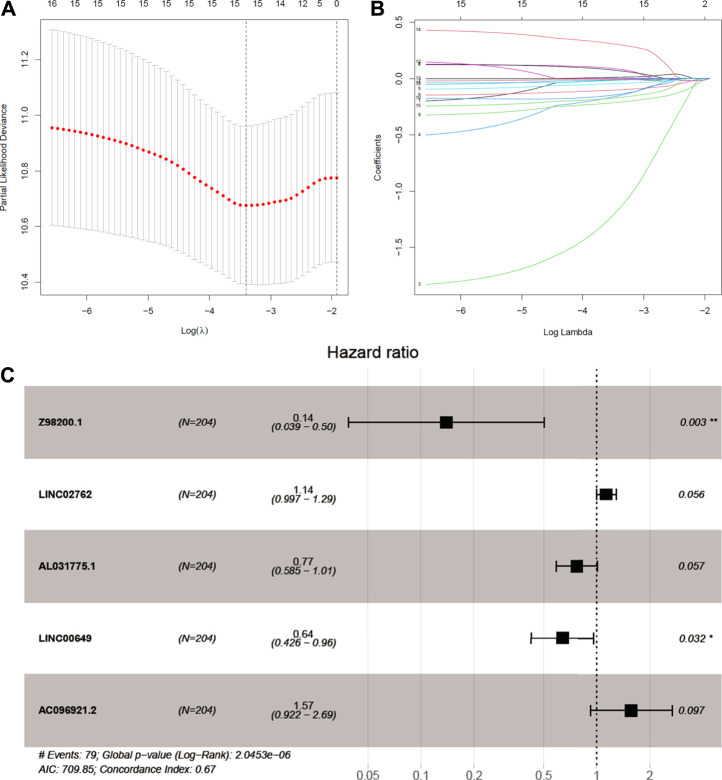
Identifcation of ferroptosis-prognosis–related lncRNAs. **(A,B)** LASSO regression analysis selected ferroptosis-prognosis–related lncRNAs. **(C)** Multivariate Cox regression analysis to identify independent risk factors for bladder cancer.

We try to discuss the relationship between ferroptosis-related lncRNAs and survival. As a result, 16 ferroptosis-related lncRNAs were closely related with OS in the univariate Cox regression analysis (Supplementary Figure S1B). We build the risk signature of the five ferroptosis-related lncRNAs by the LASSO regression analysis (Figures 3A,B). Then, the further research result showed that two of the five ferroptosis-related lncRNAs genes with hazard ratio (HR) > 1 (*AC096921.2* and *LINC02762*) could be poor prognostic markers for BC and other three genes with a HR < 1 (*Z98200.1*, *LINC00649*, and *AL031775.1*) may be protective markers (Figure 3C).

**FIGURE 4:**
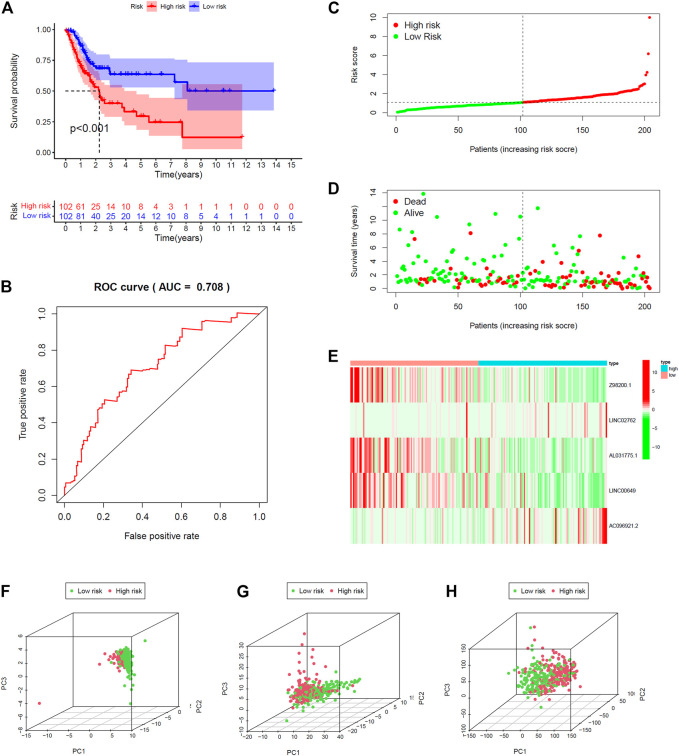
Validation of prediction model in training set. **(A)** Kaplan–Meier survival curves revealed OS comparison of the high- and low-risk groups. **(B)** Receiver operating characteristic (ROC). Distribution of risk scores **(C)** and survival status **(D)**. **(E)** Expression heatmaps of the five ferroptosis-prognosis–related lncRNAs. **(F,G,H)** Principal component analysis (PCA) was used to explore the distribution of samples in different risk group.

All 406 BC samples were then divided into training set and testing set by the complete randomization method. The training set included 204 samples, which were divided into high-risk group (*n* = 102) and the low-risk group (*n* = 102) according to the calculated median risk score. Kaplan–Meier survival curve revealed that the OS in the high-risk group was significantly lower than that in the low-risk group (*p* < 0.001; Figure 4A). We plotted ROC and the area under the curve (AUC) was 0.708 (Figure 4B). The differences between the two risk groups in the distribution of risk score and survival status were further explored. (Figures 4C,D). Patients with BC in the high-risk group had high expression of *AC096921.2* and *LINC02762* but low expression of *Z98200.1*, *LINC00649*, and *AL031775.1* (Figure 4E).

The testing set and entire set were divided into high-risk group (*n* = 109 in the testing set, *n* = 211 in the entire set) and low-risk group (*n* = 93 in the testing set, *n* = 195 in the entire set) by the same way as training set. There was significant difference in the OS rate between the two groups according to the risk score. Patients in the high-risk group had poorer OS than the low-risk group (*p* < 0.012, *p* < 0.001; Supplementary Figures 2A, 3A). The AUC was 0.656 and 0.683 in the testing set and the entire set, respectively (Supplementary Figures S2B, 3B). The distributions of the risk score and survival state were shown in Supplementary Figures S2C,D and Supplementary Figures S3C,D. The expression plotted of the five ferroptosis-related lncRNAs is demonstrated in Supplementary Figures S2E, 3E. To explore the function of the five ferroptosis-related lncRNAs in BC, we performed PCA that showed that the whole genome expression, DE ferroptosis-related lncRNAs, and the above risk genes in the model could separate the high- and low-risk groups (Figures 4F–H). The results showed that samples in the two risk groups generally had different ferroptosis states and may be identified by the lncRNA signature.

**FIGURE 5:**
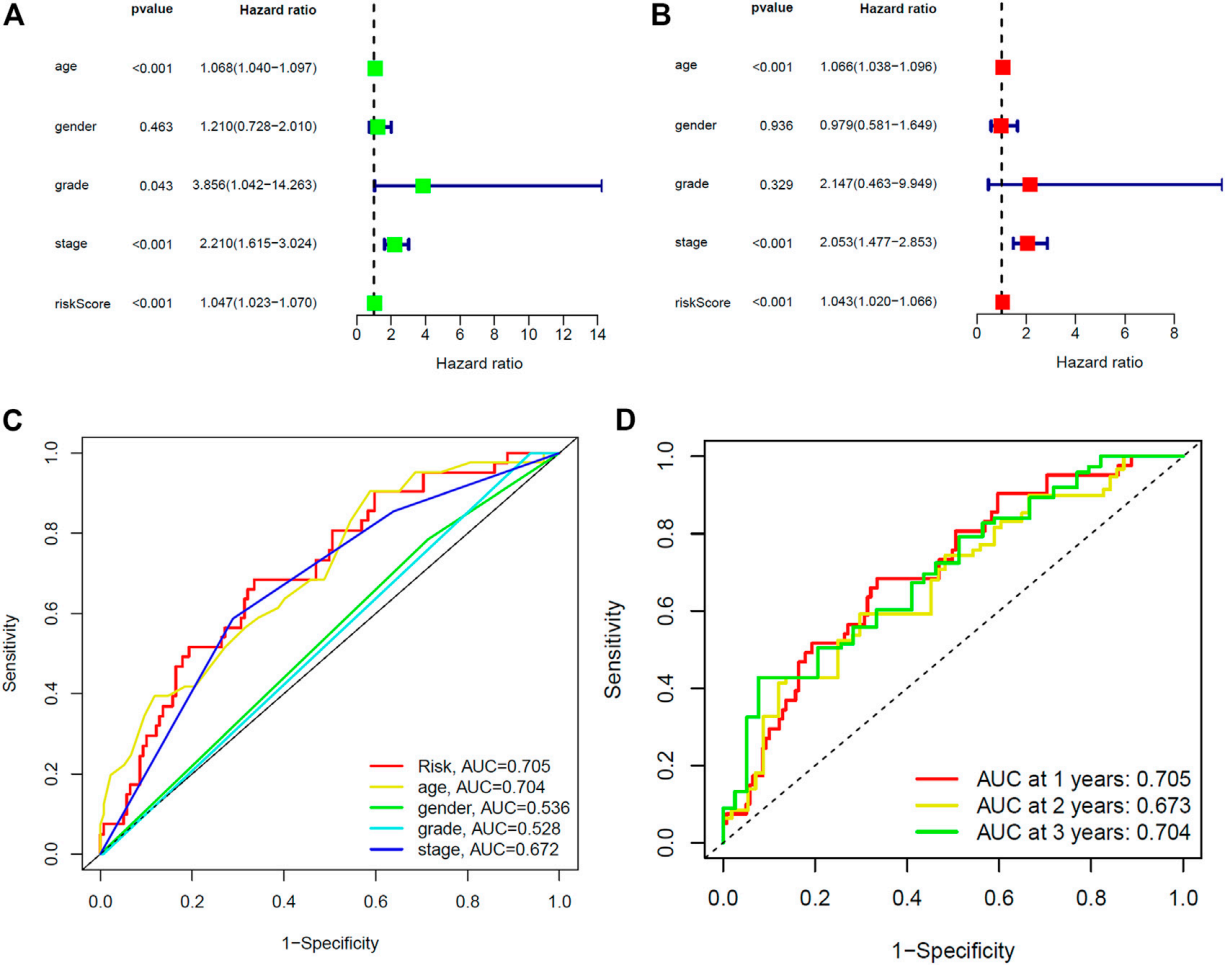
Identification of prognostic indicators for bladder cancer in training set. **(A)** Univariate Cox regression analysis showed that age, stage and the risk score were prognostic factors of bladder cancer. **(B)** Age, stage, and the risk score are independent risk factors for bladder cancer. **(C)** ROC curve of prognostic indicators for bladder cancer. **(D)** Time-dependent ROC curve of the risk score.

Furthermore, we compared the prognostic relevance of BC between the risk score and clinical variables (age, gender, grade, and stage). Univariate Cox regression analysis in the training and entire sets revealed that age, stage, and the risk score were directly related to the prognosis of BC (Figure 5A, Supplementary Figure S5A; *p* < 0.001). In addition, the multivariate Cox regression analysis showed that age, stage, and the risk score were independent prognostic indictors in BC (Figure 5B, Supplementary Figure S5B; *p* < 0.001). The AUC corresponding to the risk score (training set, 0.705; testing set, 0.652; entire set, 0.680) was higher than that for age (training set, 0.704; testing set, 0.610; entire set, 0.660), gender (training set, 0.536; testing set, 0.411; entire set, 0.479), grade (training set, 0.528; testing set, 0.527; entire set, 0.528), and stage (training set, 0.672; testing set, 0.600; entire set, 0.639) (Figure 5C, Supplementary Figures S4C, 5C). This risk score was an independent risk factor for BC prognosis and had good predictive power at 1, 2, and 3 years (Figure 5D, Supplementary Figures S4D, 5D). The results demonstrated that the risk score based on five lncRNAs may predict the OS rate of patients with BC.

### Interaction Network

**FIGURE 6:**
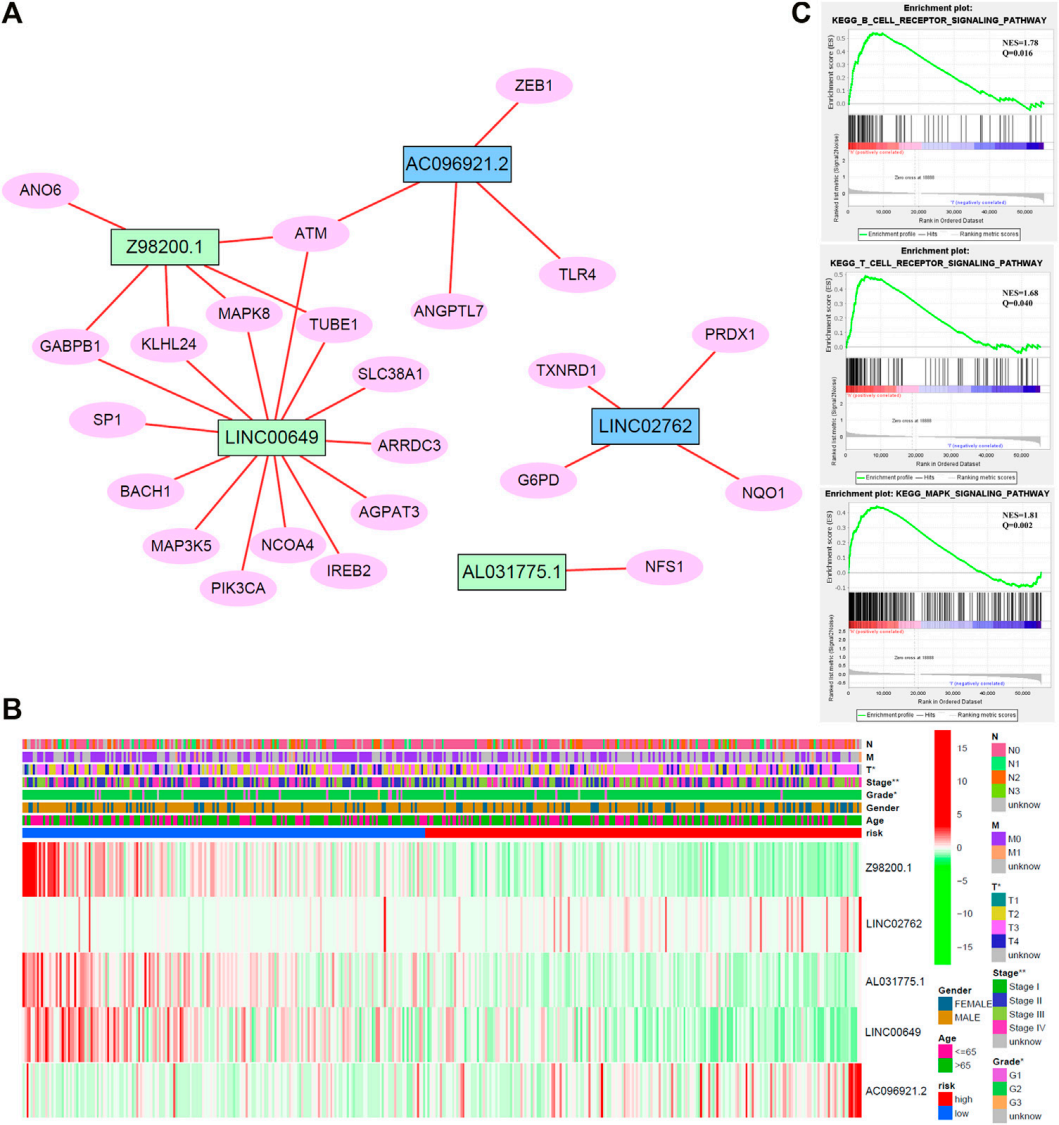
**(A)** Network diagram of five target lncRNA-related genes. **(B)** Cluster analysis. **(C)** Gene set enrichment analysis (GSEA) of target lncRNAs.

To enhance the understanding of potential interactions among the five ferroptosis-related lncRNAs, a total of 23 related genes were obtained by gene coexpression analysis. Furthermore, Cytoscape software was used to construct the network (|cor|>0.3, *p* < 0.05; Figure 6A). *ATM* may be a junction of the genes network, which connected *Z98200.1*, *LINC00649*, and *AC096921.2*.

### Comparative Analysis of Different Risk Groups

Cluster analysis of stage, grade, TNM stage, age, gender distribution, and target gene showed that patients with worse pathological stage, higher grade, and later stage had higher risk score, higher expression of *AC096921.2* and *LINC02762*, and lower expression of *Z98200.1*, *LINC00649*, and *AL031775.1* (Figure 6B). Patients in the high-risk group had a later clinical stage than those in the low-risk group. T stage, gender, and stage were closely related to the risk score.

In addition, our previous work revealed that m6A played an important role in lung cancer (Yin et al., 2021). Hence, we explored the expression of m6A methylation gene in patients with BC. There were obvious differences between the high- and low-risk groups. Expression of *YTHDF1*, *YTHDF2*, *YTHDC1*, and *METTL3* in the low-risk group was significantly high compared with the high-risk group, which suggests that m6A modification may affect the progression of BC (Supplementary Figure S6A).

### The Target lncRNAs May Be Bound up With Immunity

To study the biological pathways and functions involved in the pathogenesis of BC, we performed gene set enrichment analysis. The five lncRNAs may participate in B-cell receptor signaling pathway, T-cell receptor signaling pathway, and MAPK signaling pathway (Figure 6C).

**FIGURE 7:**
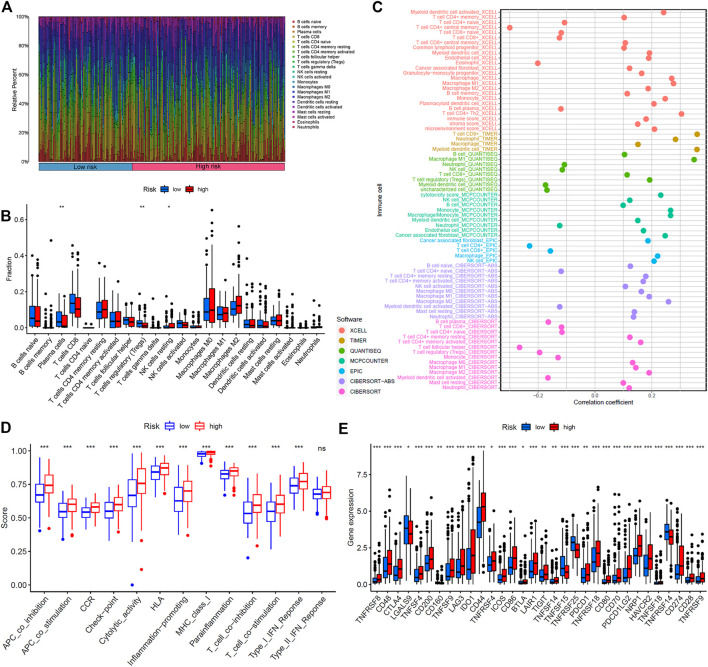
Immunoassay showed that the five ferroptosis-prognosis–related lncRNAs were closely related to the immune system. **(A)** Proportion of immune cells in the high- and low-risk groups. **(B)** Immune cell abundance in the high- and low-risk groups. **(C)** Correlation analysis between immune cell abundance and the risk score. **(D)** Immune function analysis showed that there were significant differences between the high- and low-risk groups. **(E)** Comparison of immune checkpoints between two risk groups.

**FIGURE 8:**
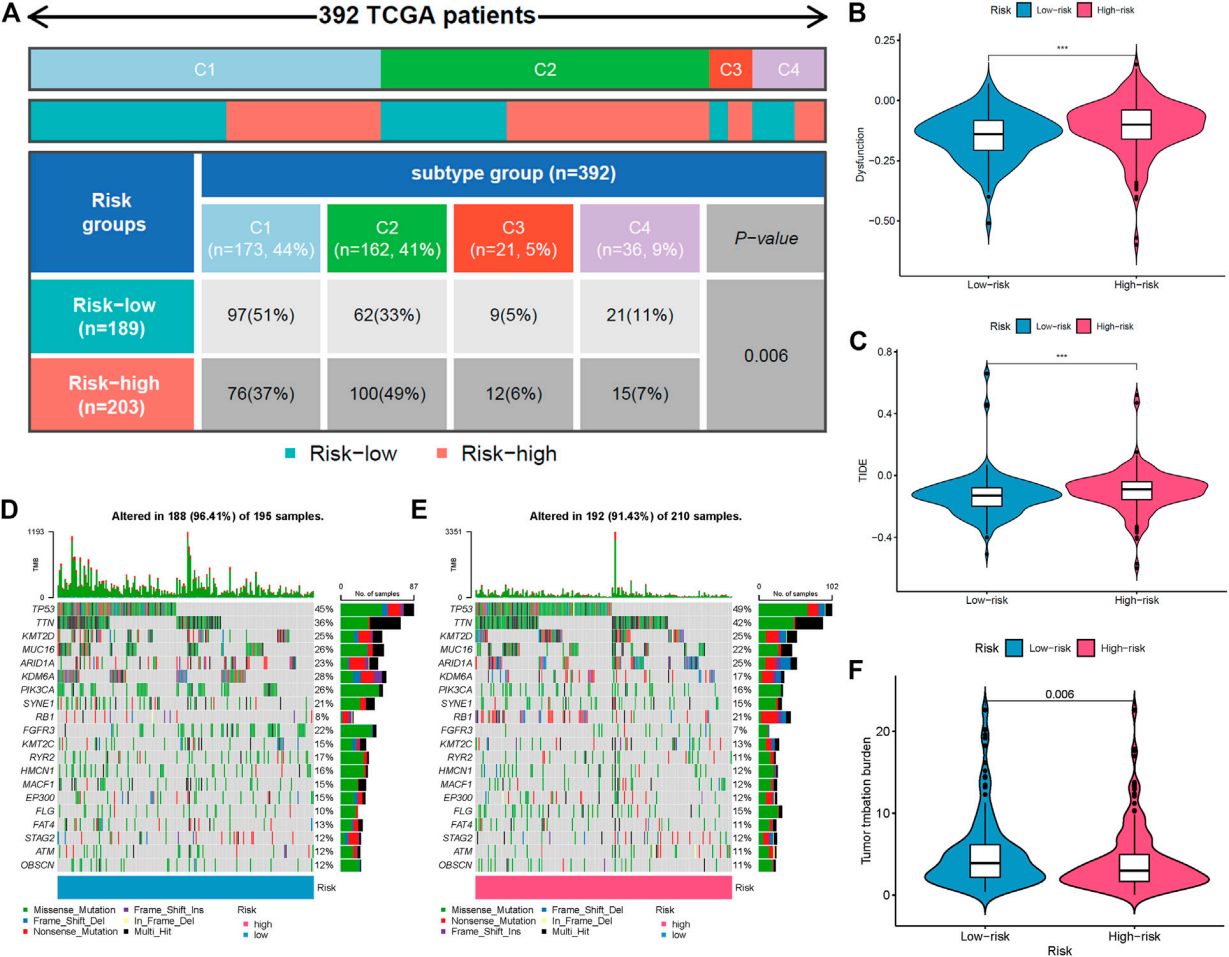
Comparison of two risk groups in molecular typing of immune subtypes, tumor mutation burden (TMB), and immune escape. **(A)** Proportion of molecular typing of immune subtypes. **(B)** The high-risk group had more immune dysfunction. **(C)** Compared with the low-risk group, the high-risk group had higher Tumor Immune Dysfunction and Exclusion (TIDE) score and greater potential of immune escape. **(D)** TMB of the low-risk group. **(E)** TMB of the high-risk group. **(F)** Statistical analysis of TMB in two risk groups.

The immune response to tumor affects the progress of tumor to a great extent. Immune cell infiltration in tumor microenvironment (TME) can reflect the immune response to tumor. The proportion of immune cells in the two risk groups is shown in Figures 7A,B. There were distinct differences in immune response between the high- and low-risk groups. The immune cell abundance was significantly correlated with the risk score (Figure 7C). In addition, the analysis of immune score showed that the immune function of the high-risk group was stronger than that of the low-risk group (Figure 7D). We further explored the immune checkpoint genes in these groups. The results revealed that the genes were generally highly expressed in the high-risk group except *LGALS9*, *TNFSF15*, *TNFRSF25*, and *TNFRSF14* (Figure 7E). The mechanism of the five lncRNAs may be related to immune response. Furthermore, type C1 (wound healing) immunization is the main immunization mode in the low-risk group, whereas type C2 [interferon-γ (IFN-γ) dominant] immunization accounts for a high proportion in the high-risk group (Figure 8A). The proportion of type C3 (inflammatory) and type C4 (lymphocyte depleted) immunization was low in the two groups. The difference of molecular typing of immune subtypes between the two risk groups was statistically significant. Moreover, the high-risk group had higher TIDE prediction scores and patients may not benefit from immune checkpoint inhibitor (ICI; Figures 8B,C). Programmed death inhibitor-1 (PD-1) protein or its ligand (PD-L1) plays an important role in many tumor treatments. TMB is an index to evaluate the efficacy of PD-1 antibody therapy. The analysis showed that the low-risk group has a lower TMB as a whole (Figures 8D,E). Therefore, its immunotherapy response rate may be higher. The difference of TMB was statistically significant (Figure 8F).

The low-content group of memory B cells (*p* = 0.012), macrophages (M0: *p* = 0.014; M2: *p* < 0.001), resting mast cells (*p* = 0.018), neutrophils (*p* < 0.001), and activated natural killer cells (*p* = 0.032) had better OS. For plasma cells (*p* < 0.001), activated CD4^+^ memory T cells (*p* = 0.001), and CD8^+^ T cells (*p* < 0.001) (Supplementary Figures S7A–F), better survival appeared in the high-content group (Supplementary Figures S7G–I). In addition, the survival analysis of immune function showed that there were significant differences in survival between immune score groups, and the high–immune score group had better OS than the low–immune score group (Supplementary Figure S8).

### Significance of the Risk Model in Routine Chemotherapy

**FIGURE 9:**
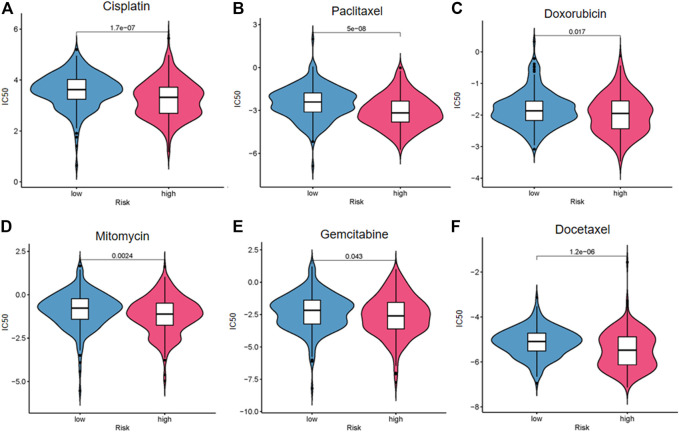
Drug sensitivity analysis of cisplatin **(A)**, paclitaxel **(B)**, doxorubicin **(C)**, mitomycin **(D)**, gemcitabine **(E)**, and docetaxel **(F)**.

We explored the response of patients with BC with different risk scores to conventional chemotherapy drugs including cisplatin, paclitaxel, doxorubicin, mitomycin, gemcitabine, and docetaxel (Figure 9). The difference of drug sensitivity between the high- and low-risk groups was statistically significant. Patients in the high-risk group had higher drug sensitivity than those in the low-risk group. The findings suggest that this risk model may be helpful for clinical treatment and prevention of drug resistance in patients with BC.

### Single Gene Risk Analysis

**FIGURE 10:**
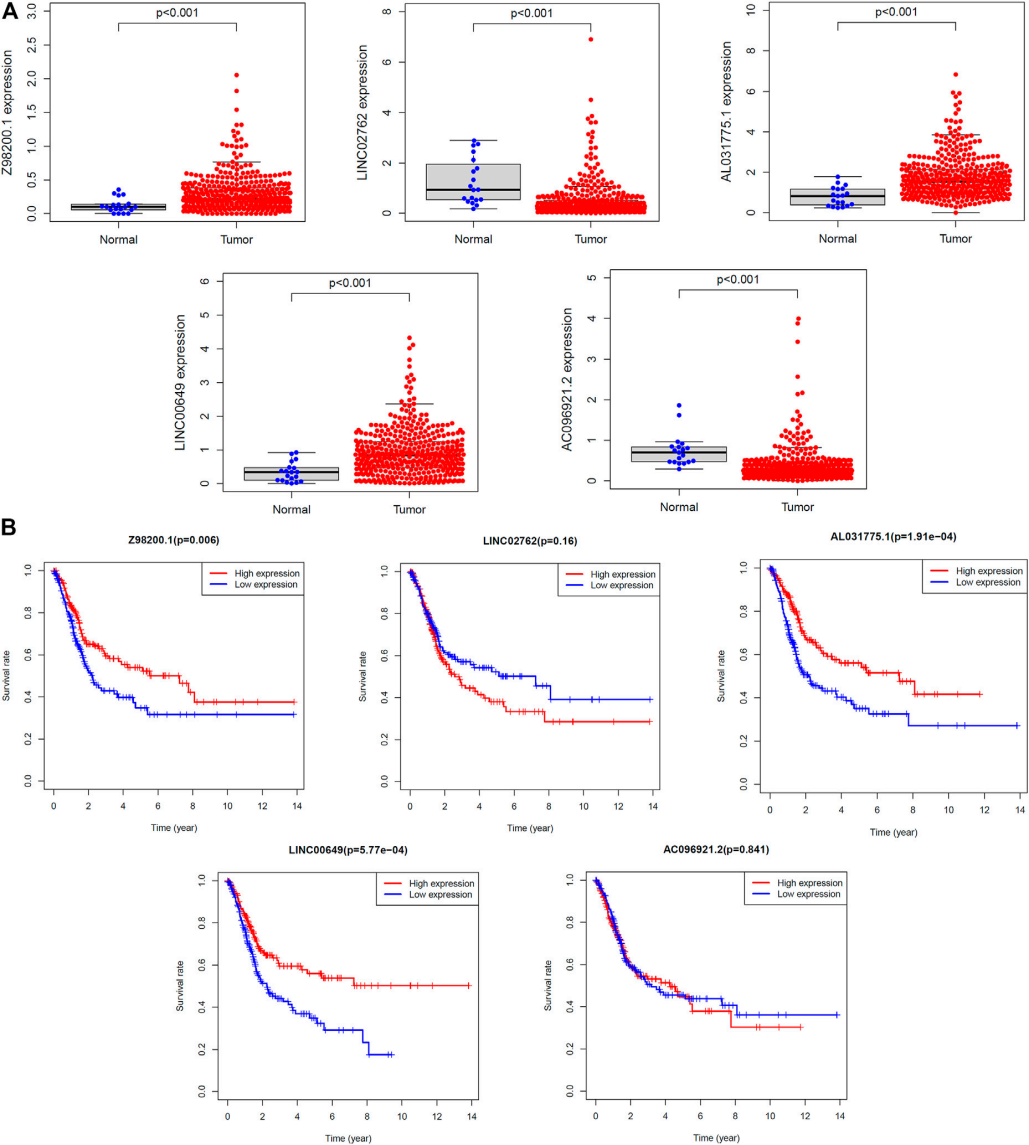
Survival analysis of single lncRNA. **(A)** The expression levels of five target lncRNAs in tumor tissues and normal tissues were compared. **(B)** The samples were grouped according to the median expression size for survival analysis, and the survival analysis was carried out.

The diversity in expression of the five ferroptosis-related lncRNAs genes between BC tissues and normal tissues was shown in Figure 10A. The 406 patients with BC were divided into high- and low-expression groups according to the median expression level of each lncRNA. The Kaplan–Meier curve of the high- and low-expression groups showed that *Z98200.1*, *LINC00649*, and *AL031775.1* were positively correlated with the prognosis of BC and that *LINC02762* was negatively correlated with the prognosis of BC (Figure 10B).

## Discussion

Some research studies reported the change characteristics of morbidity and mortality of BC from 1990 to 2016 and predicted that the morbidity will continue to rise by 2030, especially in the high social population index countries (Cai et al., 2020). European Organization for Research and Treatment of Cancer divided patients with BC into low-, medium-, and high-risk groups according to tumor size, number, T category, recurrence rate, *in situ* cancer, and grading (Sylvester et al., 2006). Hence, the discovery and application of more prognostic predictors in BC may improve the survival rate of patients.

Increasing studies have confirmed that lncRNAs potentially participate in cancer progression. Our current study explored the differential expression of ferroptosis-related ncRNA genes in patients with BC and found 463 upregulated genes and 75 downregulated genes. LncRNAs may affect the occurrence and progression of BC through ferroptosis. The results indicate that lncRNAs could regulate development of cancer on many levels such as the TME, tumor growth, invasion, metastasis, and recurrence (Statello et al., 2021). Focally amplified lncRNA on chromosome 1 (*FAL1*) repressed P21 to regulate the cell proliferation (Hu et al., 2014). Gastric cancer–associated lncRNA1 (*GClnc1*) may promote progression of BC *via* activation of *MYC* (Zhuang et al., 2019). Although many studies have been devoted to the role of lncRNAs in tumors, further research in the prognosis of BC is still extremely needed.

Further analysis showed that these genes may play a role by affecting microRNAs and PI3K-Akt signaling pathway. The PI3K pathway was widely activated in BC, which could be the potential therapeutic targets (Cancer Genome Atlas Research 2014). Pictilisib (an effective PI3K inhibitor) synergized with cisplatin and/or gemcitabine could significantly delay the growth of BC compared with single-drug treatment (Zeng et al., 2017). The inhibitor of PI3K acted synergistically with fibroblast growth factor receptor inhibitors in BC, which plays a significant role of targeted therapeutics (Wang et al., 2017). Inhibition of PI3K pathway may activate the corresponding feedback pathway and affect the therapeutic effect. How to prevent drug resistance deserves further study.

Our research showed that the five ferroptosis-related lncRNAs may act on the immune system. There were significant differences between the two risk groups in immune cell abundance, immune function, immune escape, and TMB. Patients with high risk of BC may have a stronger immune response. It was found that the degree of immune infiltration and immune molecules is related to prognosis (Efstathiou et al., 2019; Seo et al., 2021). In recent years, immunotherapy has developed rapidly and is widely used in the treatment of a variety of tumors. From the BCG, which is the first approved immunotherapy drug of BC approved by the Food and Drug Administration to adoptive immunotherapy, immune checkpoint blockades, cancer vaccines, bispecific antibodies, and oncolytic viruses, more and more immunotherapy has been used in the field of BC (Wu et al., 2021). The research of immunotherapy combined with chemotherapy is emerging one after another. Maintenance avelumab plus chemotherapy with gemcitabine plus cisplatin or carboplatin significantly prolonged the progression-free survival and the OS of patients with unresectable urothelial carcinoma (Galsky et al., 2020; Powles et al., 2020). Whether primary or metastatic tumors, ICI is beneficial to the treatment of BC (Grobet-Jeandin et al., 2021). This research suggests that, using the model to predict the risk score of patients with BC, can reflect the effect of ICI and immune response rate to some extent. Immunoassays for BC may provide more diagnostic and therapeutic options for patients. In addition, the difference between the two risk groups in the treatment response of commonly used chemotherapeutic agents for BC suggests that this model may be helpful in the selection of chemotherapy regimen and the judgment of curative effect.

Interestingly, ferroptosis plays an important role in preventing drug resistance and tumor immunity, which is perceived as a major breakthrough (Viswanathan et al., 2017; Wang W. et al., 2019). Furthermore, ferroptosis can occur in a variety of immune cells and affect the immune response, among which, T cells have an effect on ferroptosis of tumors (Xu et al., 2021). Immunotherapy activated CD8^+^ T cells downregulate SLC3A2 and SLC7A11 by releasing IFN-γ, which reduces the uptake of cystine and promotes ferroptosis. In addition, ferroptosis can enhance the antitumor effect mediated by T cells (Wang W. et al., 2019). Detecting specific ferroptosis-related biomarkers may help us diagnose and treat tumors. Some scholars have proposed that gene modification can be used to enhance immunity to tumors.

Tracing upstream of the target genes, we found that they might be modified by m6A to increase or decrease their expression in BC. M6A is a base modification behavior widely existing in mRNAs. This reversible methylation occurs at the sixth nitrogen atom of adenylate. Its regulatory factors include methyltransferase (METTL3, METTL14, WTAP, and KIAA1492), demethylase [fat mass and obesity-associated protein (FTO) and ALKBH5] and methylated reading protein (YTHDC1-2, YTHDF1-3, HNRNPA2B1, and eIF). Gene methylation occurs widely in tumors. FTO mediates m6A modification of MALAT/miR-384/MAL2 axis to promote tumorigenesis of BC (Tao et al., 2021). Studies have shown that m6A modification and PI3K-Akt signaling pathway also play a role in epithelial–mesenchymal transition (EMT) (Lin et al., 2019). Primary epithelial tumor cells mainly develop type three EMT, which enhances cell invasiveness and migration, resulting in tumor progression and metastasis (Kalluri. 2009).

Our research started with ferroptosis-related lncRNAs and explored some new biomarkers for BC. It expands the layout of BC gene expression study and provides more abundant and comprehensive support for the diagnosis and treatment of BC. Early identification and risk stratification of patients with BC at the gene level is conducive to the development of precision medicine. We can also use the ferroptosis gene specifically expressed in BC as a breakthrough to inhibit tumor resistance and relapse and explore new therapeutic targets. Tumor-related research is changing with each passing day, and its mystery is gradually revealed. In the experiment, there may be some differences between reality and expectation in terms of the comparison results of the target genes expression in tumor samples and normal samples, which may due to the insufficient sample size of normal tissues. We tried to validate our risk model with another independent database of BC data, but the database with complete information was not found.

In a word, we identified some novel biomarkers closely related to survival rate of patients with BC and generated a prediction model that has positive significance in predicting prognosis of BC. The more in-depth and detailed research of ferroptosis in BC was required, and the specific pathway of target gene on each system is still unclear. Moreover, whether the treatment targeting the five lncRNAs can improve the therapeutic effect on related chemotherapeutic drugs and immunotherapy such as ICI and reduce drug resistance deserves further study.

## Data Availability

The datasets presented in this study can be found in online repositories. The names of the repository/repositories and accession number(s) can be found in the article/Supplementary Material.

## Supplementary Material

Supplementary Figure S1
**(A)** GO analysis of ferroptosis-related genes at biological process (BP) category. **(B)** Univariate Cox regression analysis of differentially expressed ferroptosis-related lncRNAs.

Supplementary Figure S2 Validation of prediction model in testing set. **(A)** Kaplan–Meier survival curves. **(B)** Receiver operating characteristic (ROC). Distribution of risk scores **(C)** and survival status **(D)**. **(E)** Expression heatmaps of the five ferroptosis-prognosis–related lncRNAs.

Supplementary Figure S3 Validation of prediction model in entire set. **(A)** Kaplan–Meier survival curves. **(B)** Receiver operating characteristic (ROC). Distribution of risk scores **(C)** and survival status **(D)**. **(E)** Expression heatmaps of the five ferroptosis-prognosis–related lncRNAs.

Supplementary Figure S4 Identification of prognostic indicators in testing set. **(A)** Univariate Cox regression analysis. **(B)** Multivariate Cox regression analysis. **(C)** ROC curve of prognostic indicators for bladder cancer. **(D)** Time-dependent ROC curve of the risk score.

Supplementary Figure S5 Identification of prognostic indicators in entire set. **(A)** Univariate Cox regression analysis. **(B)** Multivariate Cox regression analysis. **(C)** ROC curve of prognostic indicators for bladder cancer. **(D)** Time-dependent ROC curve of the risk score.

Supplementary Figure S6 Analysis of m6A-related gene expression in two risk groups.

Supplementary Figure S7 The samples were grouped according to the abundance of immune cells, and the overall survival rate between grades was compared. Memory B cells **(A)**, M0 macrophages **(B)**, M2 macrophages **(C)**, resting mast cells **(D)**, neutrophils **(E)**, activated NK cells **(F)**, plasma cells **(G)**, activated CD4^+^ memory T cells **(H)**, and CD8^+^ T cells **(I)**.

Supplementary Figure S8 The different immune function scores of the samples were calculated, and the overall survival of the high rating group and the low rating group were compared. APC co-inhibition **(A)**, checkpoint **(B)**, cytolytic activity **(C)**, HLA **(D)**, inflammation-promoting **(E)**, MHC class I **(F)**, T-cell co-inhibition **(G)**, T-cell co-stimulation **(H)**, and type I IFN response **(I)**.

## Abbreviations

AUC, Area under the curve; BC, Bladder cancer; DE, Differentially expressed; GO, Gene Ontology; KEGG, Kyoto Encyclopedia of Genes and Genomes; PCA, Principal Component Analysis; ROC, Receiver operating characteristic; TIDE, Tumor Immune Dysfunction and Exclusion; TMB, Tumor mutation burden.
