# Supplementary figures and images for: Development and Validation of Ferroptosis-Related LncRNA Biomarker in Bladder Carcinoma
Source: Front Cell Dev Biol. 2022 Mar 2;10:809747. doi: 10.3389/fcell.2022.809747 (PMC8924052; doi:10.3389/fcell.2022.809747)

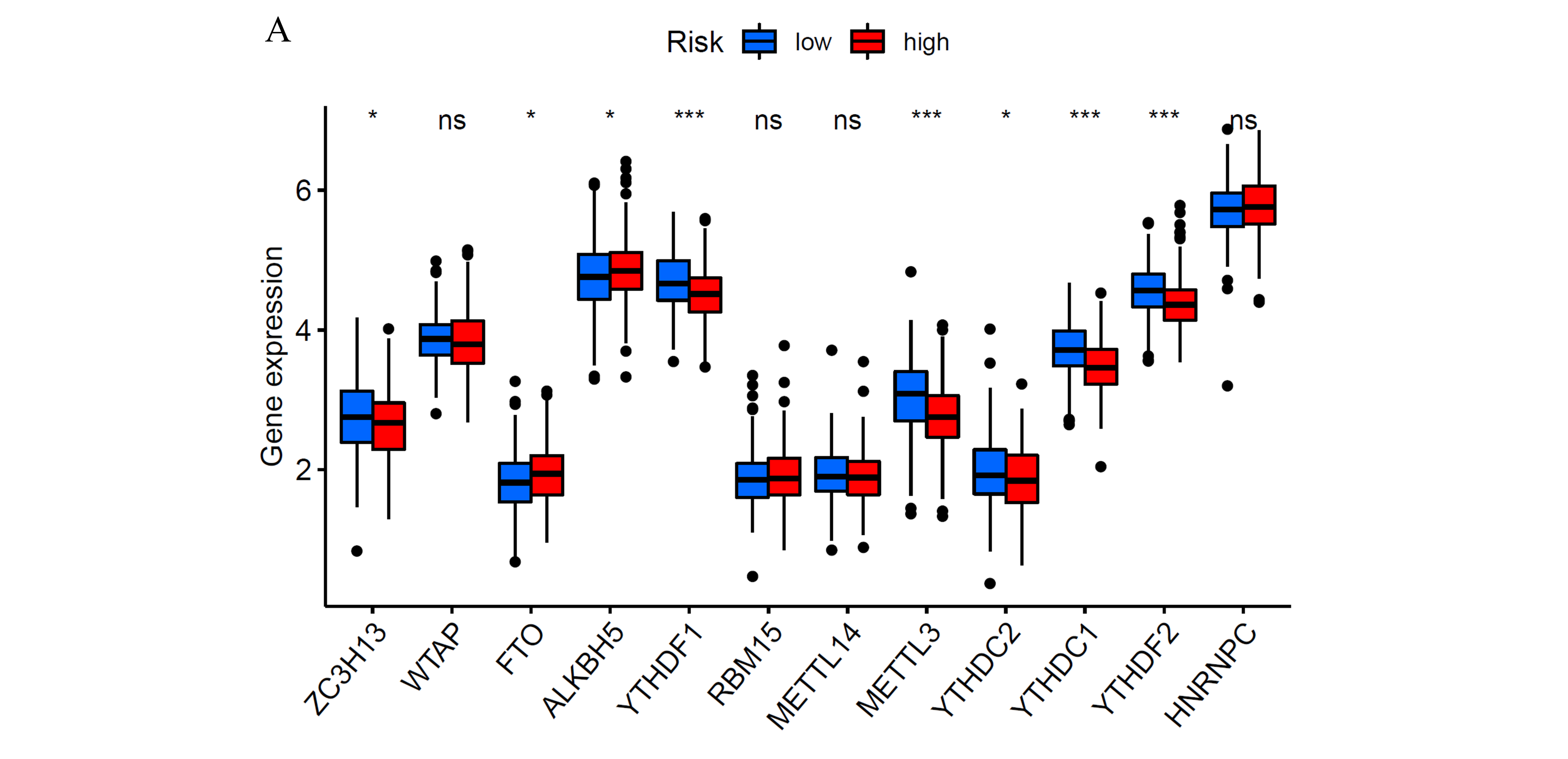

Supplement: Supplementary file 1 [file Image6.TIF]

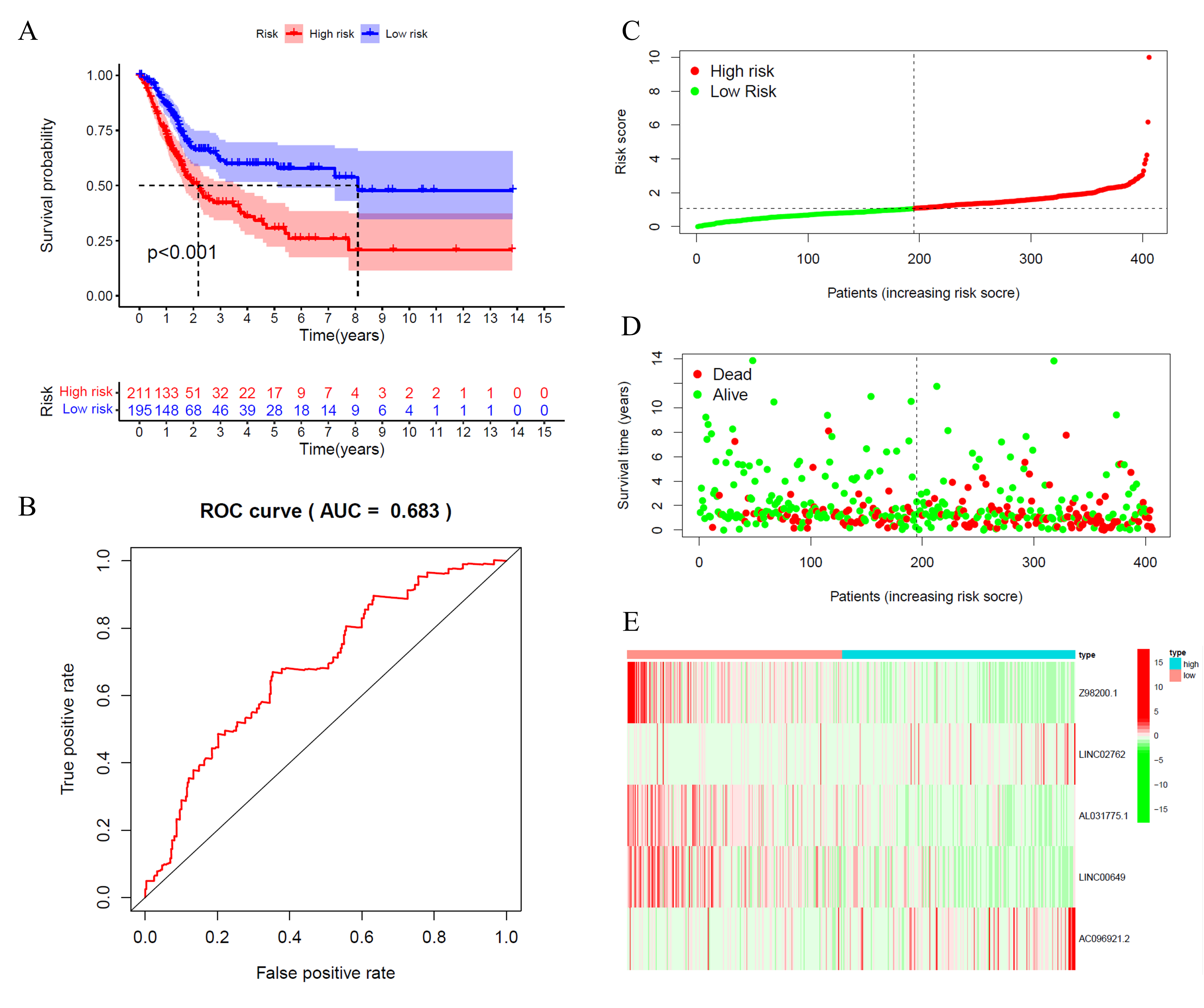

Supplement: Supplementary file 2 [file Image3.TIF]

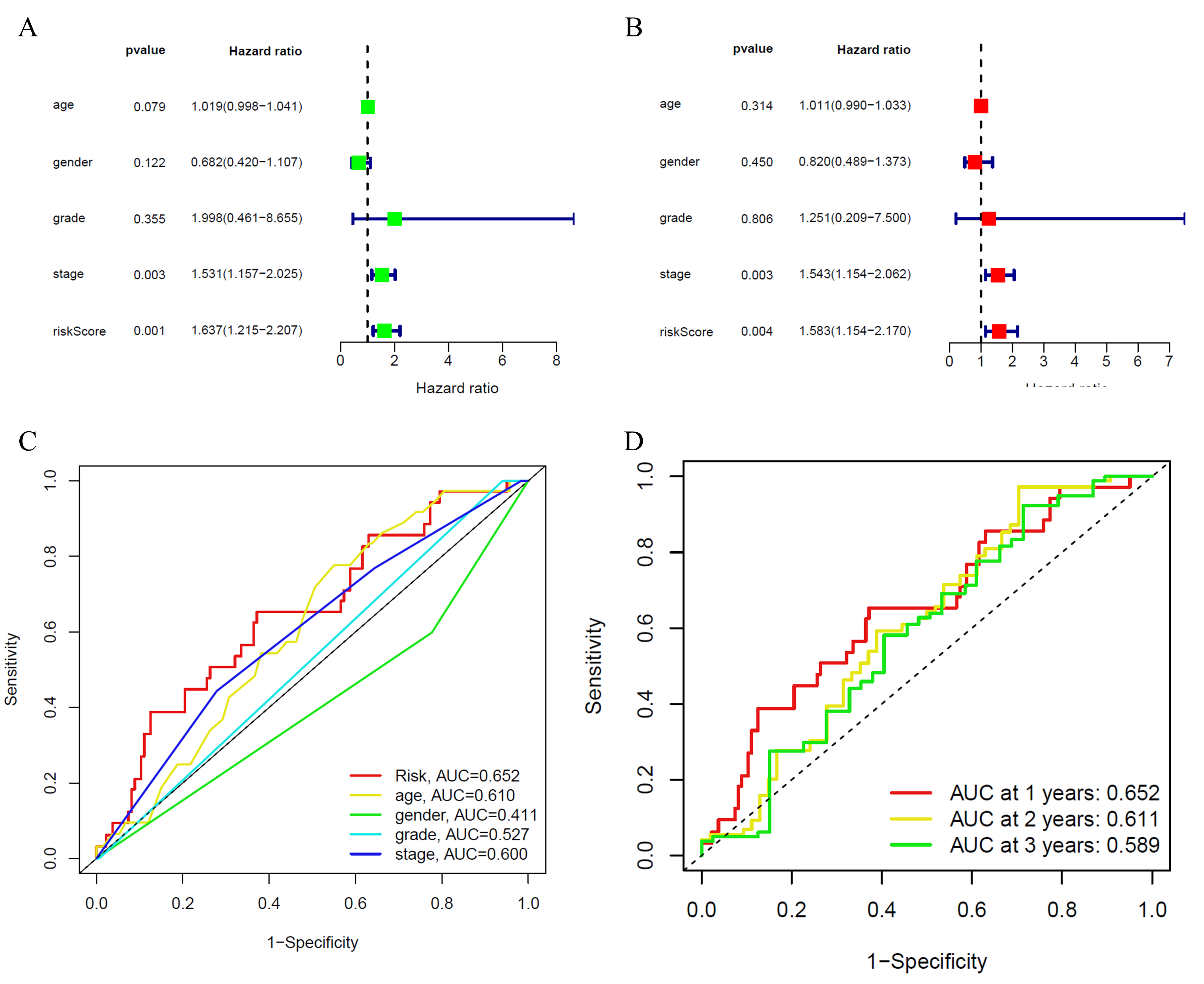

Supplement: Supplementary file 3 [file Image4.TIF]

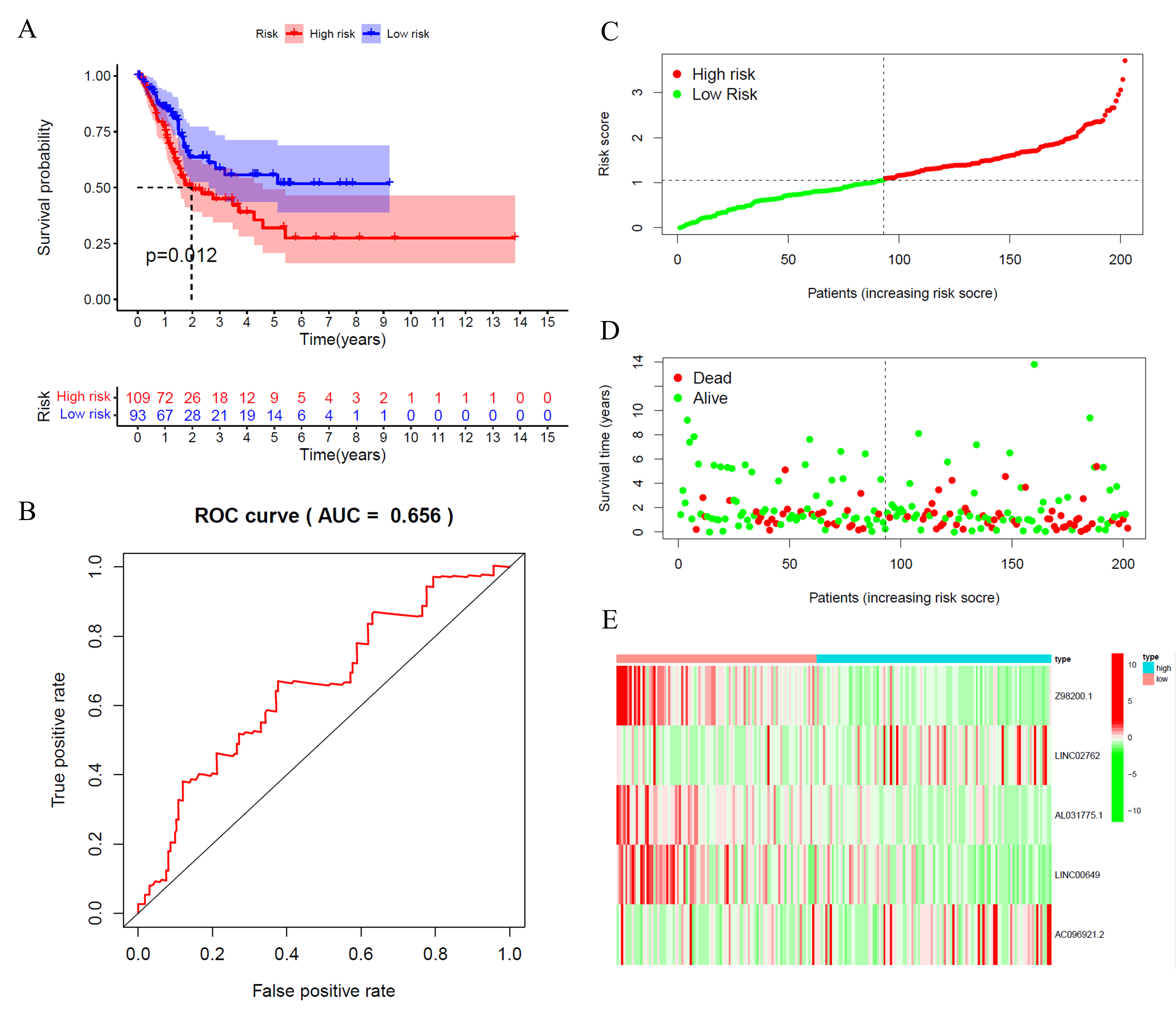

Supplement: Supplementary file 4 [file Image2.TIF]

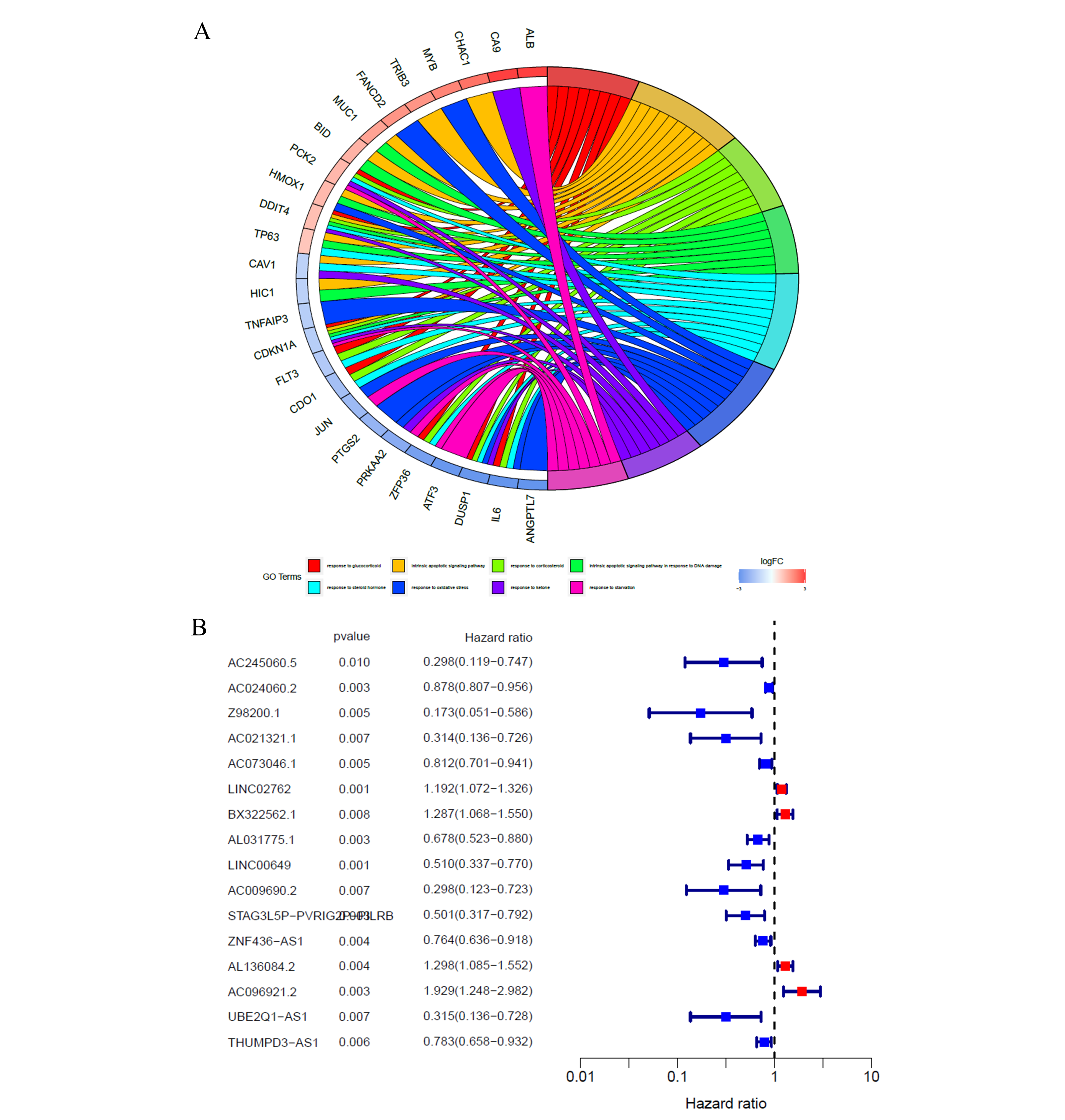

Supplement: Supplementary file 5 [file Image1.TIF]

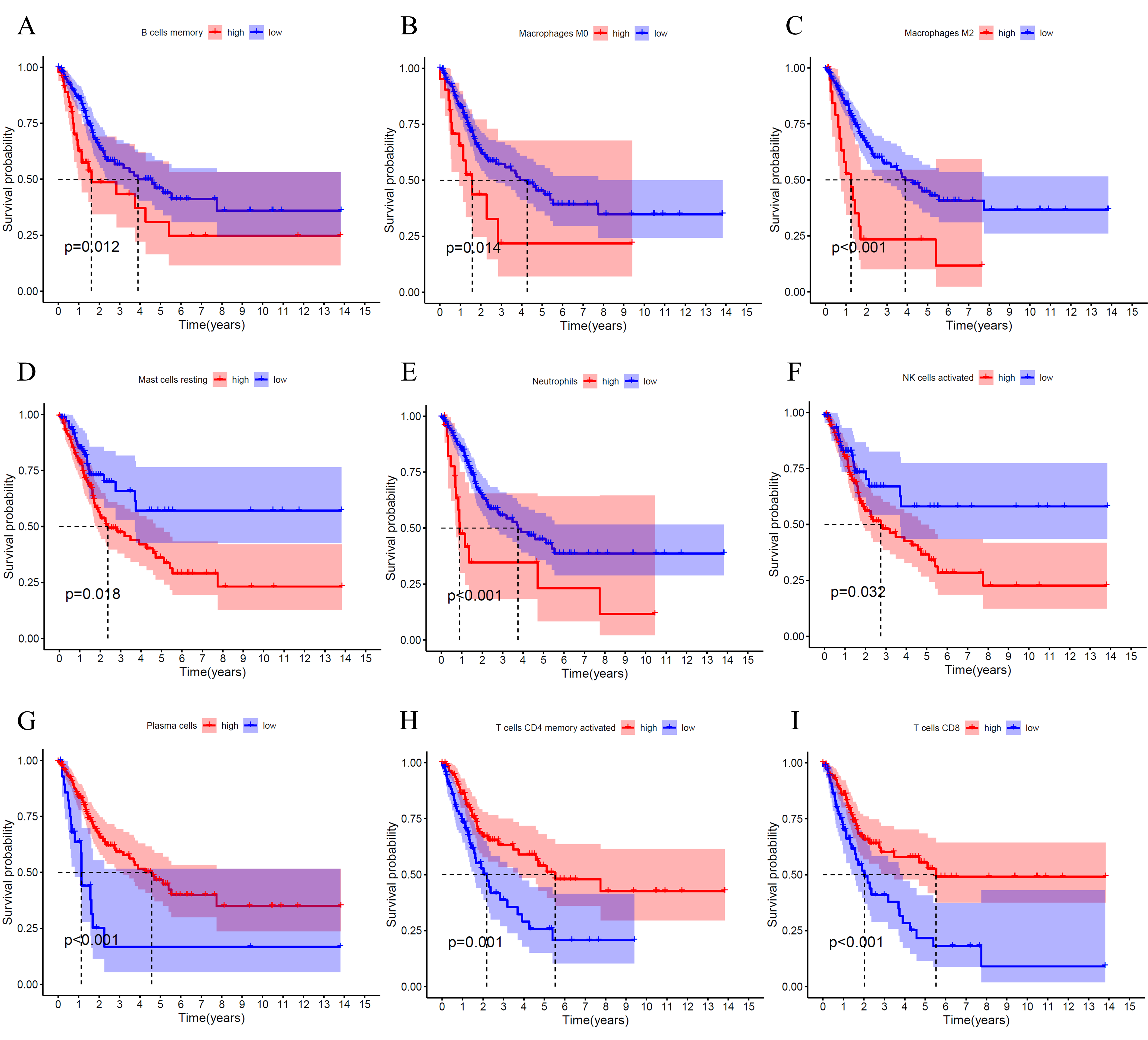

Supplement: Supplementary file 6 [file Image7.TIF]

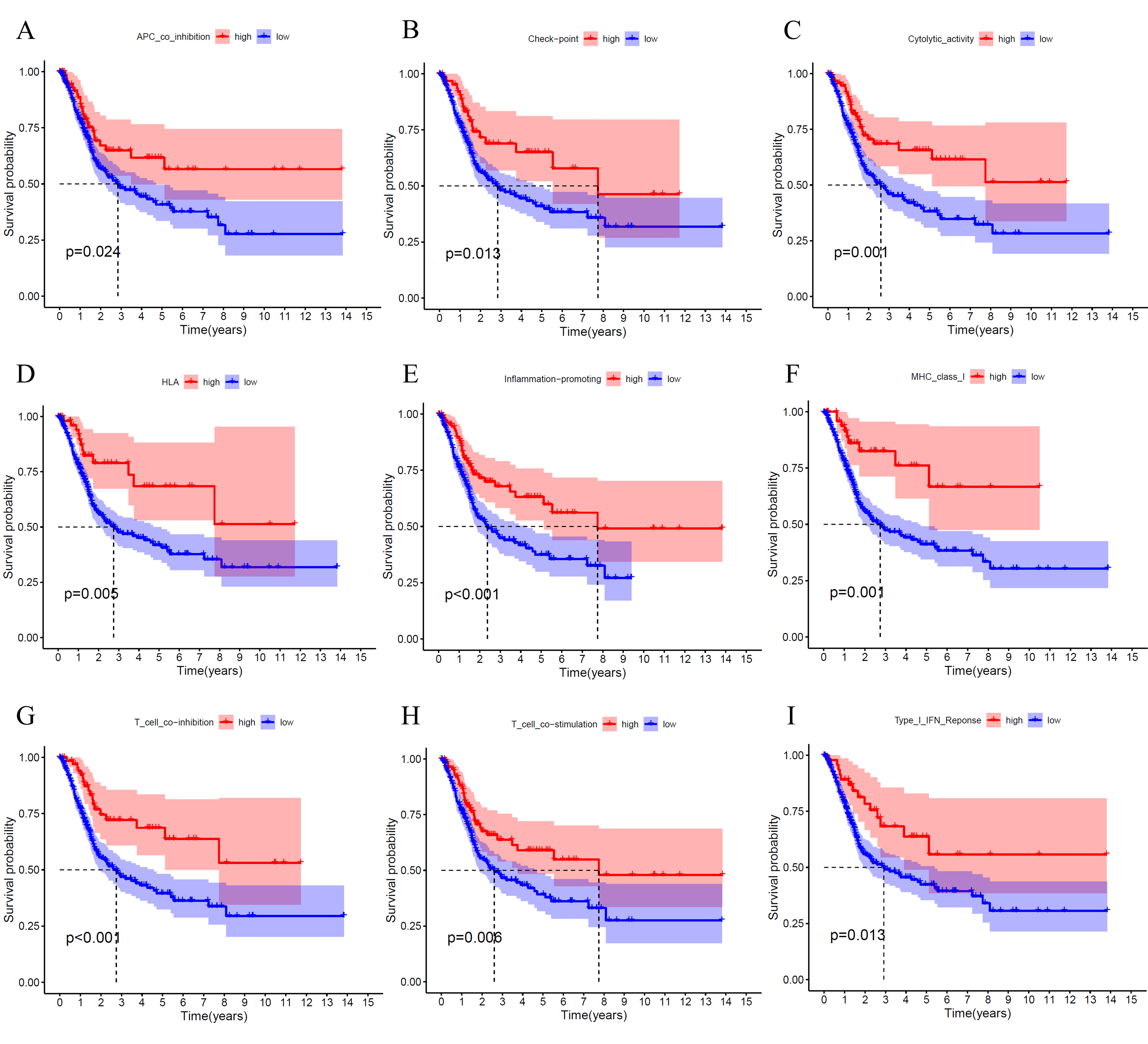

Supplement: Supplementary file 7 [file Image8.TIF]

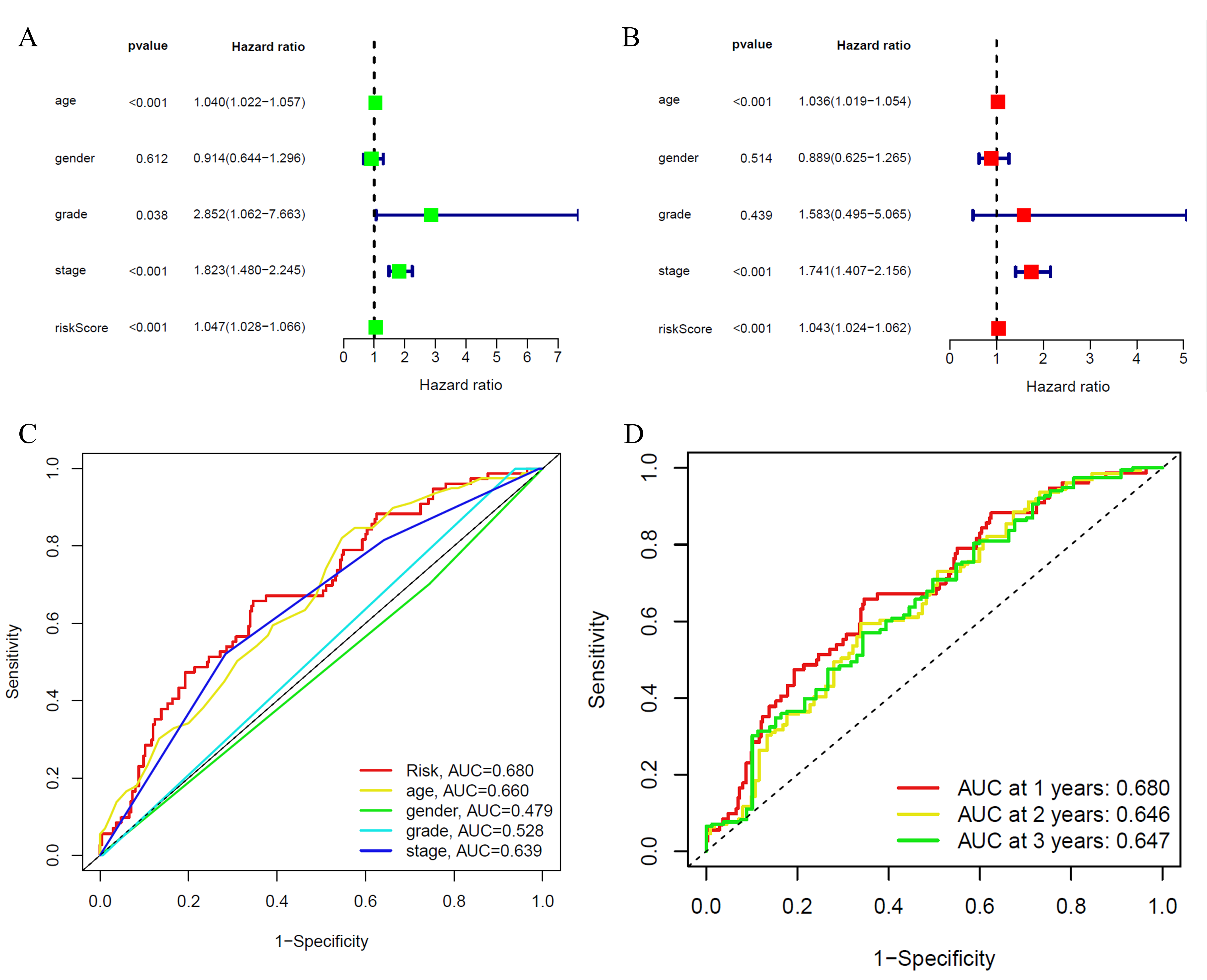

Supplement: Supplementary file 8 [file Image5.TIF]
